# Coagulant Effects and Mechanism of *Schefflera heptaphylla* (L.) Frodin

**DOI:** 10.3390/molecules24244547

**Published:** 2019-12-12

**Authors:** Xuqiang Liu, Jing Dong, Qiongxin Liang, Hui-min David Wang, Zhenhua Liu, Ruian Xu, Wenyi Kang

**Affiliations:** 1 National R & D Center for Edible Fungus Processing Technology, Henan University, Kaifeng 475004, China; liuxuqiang6230@163.com (X.L.); Dongjing@vip.henu.edu.cn (J.D.); liangqiongxin11@163.com (Q.L.); 2 Engineering Research center of Molecular Medicine, Ministry of Education, Huaqiao University, Xiamen 361021, China; 3 School of Biomedical Sciences and School of Medicine, Huaqiao University, Xiamen 361021, China; 4 Graduate Institute of Biomedical Engineering, National Chung Hsing University, Taichung City 402, Taiwan; davidw@dragon.nchu.edu.tw

**Keywords:** *Schefflera heptaphylla*, Triterpenoid, hemostasis, pro-coagulation, mechanism

## Abstract

*Schefflera heptaphylla* (L.) Frodin, are commonly used in anti-inflammatory, analgesic, traumatic bleeding and hemostasisas. In this paper, the coagulation effect of the ethanol extract (Set), ethyl acetate phase (Sea) and *n*-butanol phase (Sbu) was evaluated by prothrombin time (PT), activated partial thromboplastin time (APTT), thrombin time (TT) and fibrinogen content (FIB) assays in vitro. Then, Three main lupanine triterpenes (compounds **A**–**C**) were isolated and identified from Sea and Sbu by a bioassay-guided method and their structure were identified as 3α-Hydroxy-lup-20(29)-ene-23, 28-dioic acid, betulinic acid 3-*O*-sulfate and 3α-Hydroxy-lup-20(29)-ene-23, 28-dioic acid 28-*O*-(α-l-rhamnopyranosyl(1→4)-*O*-β-d-glucopyranosyl(1→6))-β-d-glucopyranoside) by spectroscopic data analysis. Among of them, compound **B** was confirmed to have significant coagulant effect in vitro. Furthermore, the pro-coagulation mechanism of *S. heptaphylla* extracts and compound **B** were investigated by measuring whole blood viscosity (WBV), plasma viscosity (PV), erythrocyte sedimentetion rate (ESR), pack cell volume (PCV), APTT, PT, TT, and FIB in vivo. Meanwhile, the levels of thromboxane B_2_ (TXB_2_), 6-keto prostaglandin F_1α_ (6-keto-PGF_1α_), endothelial nitric oxide synthase (eNOS) and (endothelin-1) ET-1 were detected. The bleeding time (BT) was tested by tail bleeding method, which proved the traumatic bleeding and hemostasis activities of *S. heptaphylla*. The pharmacology experiments showed that the Set, Sea, Sbu and compound **B** has significant pro-coagulation effect. In addition, compound **B** might be the main constituent of pro-coagulation in *S. heptaphylla* These results could support the fact that *S. heptaphylla* could be used traditionally to cure traumatic bleeding, and the pro-coagulation effects were associated with the regulation of vascular endothelium active substance and hemorheology parameters.

## 1. Introduction

As we all known, blood is an important substance of the human body. If the disease of bleeding is not stopped timely and effectively, it will cause blood loss and lead to weakness of the body and even endanger life, such as clinical hemorrhage, traumatic bleeding and so on. Thus, the application of hemostatic drugs is of great significance. Hemostatic agents can produce hemostatic effect by narrowing arteries and capillaries, enhancing platelet function, accelerating and strengthening blood coagulation process, or inhibiting blood clot dissolution process. Many hemostatic drugs are widely used in clinical practice, including vitamin K1, vitamin K3, vitamin K4 as activating coagulant factor; carbazochrome sodium sulfonate (CCSS), Carbazochrome as reduce capillary permeability and aminocaproic acid, aminomethylbenzoic acid as antifibrinolytic. However, hemostatic drugs, traditionally used in medical practice, are not always effective to reduce blood loss which is dangerous due to the development of hemorrhagic shock, dilutional coagulopathy, or thrombosis and thromboembolic complications [1]. It is particularly important to develop new hemostatic drugs with high efficiency and low side effects. At present, much more researchers focus on natural products, and had found some natural products with effective pro-coagulation [2,3].

Blood coagulation results from a series of proteolytic reactions involving the step-wise activation of coagulation factors I-XII. Subsets of these factors can be activated by two distinct pathways, the extrinsic and the intrinsic pathway [4,5]. The APTT is commonly used for determining the overall efficiency of the intrinsic coagulation pathway, while PT is the screening test for the extrinsic coagulation pathway [6,7]. FIB is synthesized by the liver and can be hydrolyzed by the clotting enzyme to form peptide A and peptide B, eventually form the insoluble fibrin to stop bleeding [8]. Hemorheology, used for diagnosis cardiovascular diseases in clinic, is associated with blood pressure and flow, flow volume, including WBV, PV, ESR and PCV [9,10,11]. Some factors are critical in the process of coagulation, for example, the Thromboxane A2 (TXA2) is potent vasoconstriction and platelet aggregation [12,13], which could stimulate platelet deformation and aggregation and cause the contraction of vascular smooth muscle. Prostacyclin (PGI_2_) could effectively inhibit platelet aggregation and blood vessel expansion. TXB_2_ and 6-Keto-PGF_1α_, which are mutually antagonistic, are the stable metabolites of TXA_2_ and PGI_2_, respectively [14]. The dynamic balance of TXB_2_/6-Keto-PGF_1α_ is important for the regulation of vessel wall intensity and regional flow. In addition, Nitric oxide (NO) is an important indicator of a variety of diseases and has many biological activities, including promoting inflammation, dilating blood vessels, inhibition of regulating blood pressure, platelet aggregation, dilate blood vessels and prevention of thrombosis [15,16,17]. And the eNOS could promote the continued release of NO [18,19]. In the cardiovascular systems, ET-1 is also an important factor to promote vasoconstriction, which has biological activity opposite to NO [20]. ET/NO system plays an important role in maintaining the homeostasis of the cardiovascular system [21].

*Schefflera heptaphylla* (L.) Frodin has been used as a traditional medicine for the treatment of inflammation, rheumatism, fever, traumatic bleeding [22,23,24,25,26]. Triterpenoids are the major and bioactive constituents in *S. heptaphylla* [27,28,29,30,31,32,33]. Pharmacological researches show that *S. heptaphylla* has various kinds of biological activities, such as anti-inflammatory [34], antimicrobial, antitumor [35], antiviral [36,37,38]. Although, the leaves of *S. heptaphylla* have been widely used for traumatic hemorrhage in China, there are no reports on screening of pro-coagulant ingredients from *S. heptaphylla*.

**Figure 1:**
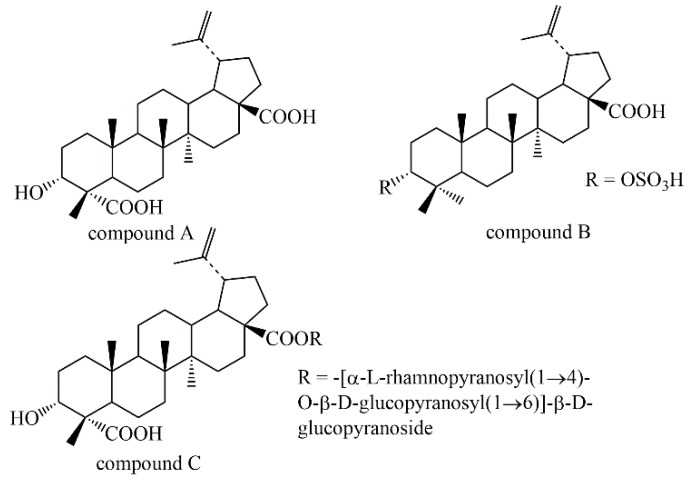
Chemical structures of compounds **A**–**C**.

Considering the traditional uses in traumatic bleeding and hemostasis of leaves of *S. heptaphylla*, we evaluated the pro-coagulation effect of Set, Sea and Sbu by Plasma coagulation parameters (PT, APTT, TT, FIB) assay in vitro. Then, three major terpenoids (compound **A**–**C**) (Figure 1), which were the main components of *S. heptaphylla*, accounting for 30% of the total content, were isolated and identified from Sea and Sbu. The clotting effect of compound **A**–**C** were invested by Plasma coagulation parameters (PT, APTT, TT, FIB) assay in vitro, indicating that compound **B** had significant pro-coagulant effect. In order to elucidate their pro-coagulation mechanism, the hemorheology indexs (WBV, PV, ESR and PCV), coagulation pathway and some key parameters (the levels of TXB_2_, 6-keto-PGF_1α_, eNOS and ET-1) were measured in heparin model rats in vivo.

## 2. Results

### 2.1. Isolation and Characterization

Three lupanine triterpenes, named compounds **A**–**C** (3α-Hydroxy-lup-20(29)-ene-23, 28-dioic acid, betulinic acid 3-*O*-sulfate and 3α-Hydroxy-lup-20(29)-ene-23, 28-dioicacid, 28-*O*-(α-l-rhamnopyranosyl(1→4)-*O*-β-d-glucopyranosyl(1→6))-β-d-glucopyranoside), were isolated from the leaves of *S. octophylla*. Their structures were determined on the basis of spectroscopic analysis and chemical methods.

*3α-Hydroxy-lup-20(29)-en-23, 28-dioic acid* (compound **A**), ESI-MS *m*/*z*: 486[M]^+^ (C_30_H_46_O_5_), ^1^H-NMR (pyridine, 400 MHz) *δ* 0.92 (3H, s, H3-27), 0.94 (3H, s, H3-25), 1.12 (3H, s, H3-26), 1.46 (3H, s, H3-24),1.77 (3H, s, H3-30), 4.31 (br.s, H-3), 4.77 and 4.94 (2× br.s, H 2 -29); ^13^C-NMR (400 MHz, pyridine, δ, ppm): 179.90 (C-23), 179.23 (C-28), 151.67 (C-21), 110.33 (C-29), 73.35 (C-3), 56.97 (C-17), 52.38 (C-4), 51.36 (C-9), 50.05 (C-18), 48.14 (C-19), 45.31 (C-5), 43.31 (C-14), 42.10 (C-8), 38.97 (C-13), 37.93 (C-10), 37.80 (C-22), 35.09 (C-7), 33.34 (C-1), 33.18 (C-16), 31.54 (C-21), 30.60 (C-15), 26.59 (C-12), 26.44 (C-2), 22.18 (C-11), 21.40 (C-6), 19.76 (C-30), 18.35 (C-24), 17.10 (C-25), 17.08 (C-26), 15.13 (C-27) [39].

*Betulinic acid 3-O-sulfate* (compound **B**), ESI-MS *m*/*z*: 536 [M]^+^ (C_30_H_47_O_6_S), ^1^H-NMR (400 MHz, DMSO-*d* 6, δ, ppm): 11.90 (1H, br.s, 28-COOH), 4.70 (1H, br.s, H-29a), 4.56 (1H, br.s, H-29b), 4.46 (1H, m, H-3), 0.79, 0.88, 1.00, 1.05, 1.26, 1.66 (each 3H, s, tert-CH 3); ^13^C-NMR (400 MHz, MeOD) δ 152.00 (C-20), 110.17 (C-29), 85.66 (C-3), 57.51 (C-17), 54.78 (C-5), 51.63 (C-9), 51.10 (C-19), 50.43 (C-18), 43.63 (C-14), 42.05 (C-8), 39.61 (C-4), 38.46 (C-13), 38.22 (C-1), 38.14 (C-22), 35.42 (C-10), 34.83 (C-7), 33.34 (C-16), 31.69 (C-15), 30.75(C-21), 28.93(C-23), 26.86 (C-12), 23.68 (C-2), 22.38 (C-11), 21.88 (C-30), 19.52 (C-6), 19.07 (C-24), 16.68 (C-25), 16.62 (C-26), 15.09 (C-27) [21].

*3α-Hydroxy-lup-20(29)-ene-23,28-dioic acid 28-O-[α-l-rhamnopyranosyl (1→4)-O-β-d-glucopyranosyl(1→6)]-β-d-glucopyranoside* (compound **C**), ESI-MS *m*/*z*: 952 [M]^+^ (C_48_H_72_O_19_), ^1^H-NMR (pyridine, 400 MHz, δ, ppm): 5.50, 5.48, 4.76 and 4.63 (2× br.s, H_2_-29), 4.40 (br.s, H-3), 1.72 (3H, s, H_3_-30), 1.15 (3H, s, H_3_-24), 1.06 (3H, s, H_3_-26), 0.98 (3H, s, H_3_-25), 0.92 (3H, s, H_3_-27); ^13^C-NMR (MeOD, 400 MHz, δ, ppm): 33.66 (C-1), 26.06 (C-2), 73.62 (C-3), 52.28 (C-4), 45.94 (C-5), 22.24 (C-6), 35.08 (C-7), 42.50 (C-8), 51.87 (C-9), 38.04 (C-10), 21.83 (C-11), 26.81 (C-12), 39.36 (C-13), 43.72 (C-14), 30.79 (C-15), 32.85 (C-16), 57.96 (C-17), 50.55 (C-18), 48.0 (C-19), 151.74 (C-20), 31.52 (C-21), 37.61 (C-22), 176.90 (C-23), 17.94 (C-24), 17.84 (C-25), 16.89 (C-26), 15.20 (C-27), 176.30 (C-28), 110.42 (C-29), 19.54 (C-30). Glc-1: 95.23 (C-1′), 73.96 (C-2′), 79.52 (C-3′), 70.63 (C-4′), 78.22 (C-5′), 69.53 (C-6′); Glc-2: 104.50 (C-1′′), 75.27 (C-2′′), 76.84 (C-3′′), 77.99 (C-4′′), 76.67 (C-5′′), 61.89 (C-6′′); Rha: 102.89 (C-1′′′), 72.39 (C-2′′′), 72.18(C-3′′′), 73.72 (C-4′′′), 70.94 (C-5′′′), 17.10 (C-6′′′) [40].

### 2.2. The Pro-Coagulant Effect of S. heptaphylla Extracts In Vitro

#### 2.2.1. Effects on Plasma Coagulation Parameters In Vitro

**Figure 2:**
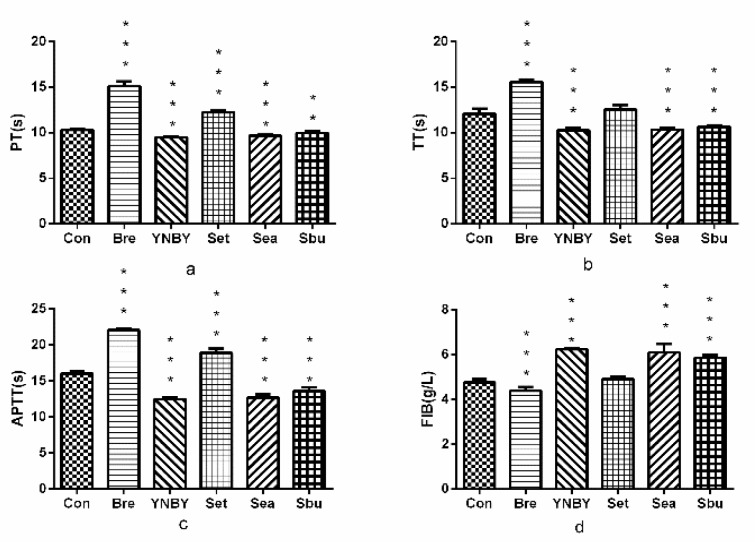
Effect on plasma coagulation parameters ((**a**). APPT; (**b**). PT; (**c**). TT; (**d**). FIB. *n* = 3, ^***^ *p* < 0.001, 0.001 < ^**^ *p* < 0.01 vs. control group.

As shown in Figure 2, Sea and Sbu could significantly shorten TT, PT, APTT (*p* < 0.01, *p* < 0.001), and significantly increase the content of FIB (*p* < 0.001) compared with the control group. However, these effects were not as obvious as compared with Yunnan abaaiyao (YNBY) (*p* > 0.05). The results showed that both of Sea and Sbu had significant pro-coagulant activity.

#### 2.2.2. The Coagulation Effect of Compound **A**–**C** in vitro

**Figure 3:**
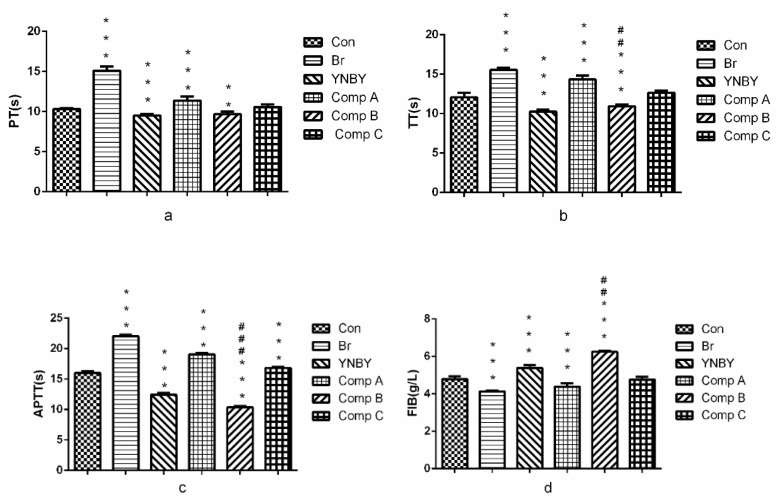
Effect on plasma coagulation parameters (**a**. PT; **b**. TT; **c**. APPT; **d**. FIB. *n* = 3, ^***^ *p* < 0.001, 0.001 < ^**^ *p* < 0.01 vs. control group, ^###^ *p* < 0.001, 0.001 < ^##^ *p* < 0.01 vs. YNBY group).

In Figure 3, compound **A** could significantly prolong PT, APTT, TT (*p* < 0.001) and decrease the content of FIB (*p* < 0.001) compared with the control group, which indicated that compound **A** had anti-coagulation effect. However, compound **B** could significantly shorten PT, APTT, TT (*p* < 0.01, *p* < 0.001, *p* < 0.001, respectively) and increase the content of FIB (*p* < 0.001) compared with the control group. APTT, TT and FIB of compound **B** were better than that of YNBY (*p* < 0.01, *p* < 0.001, *p* < 0.001, respectively). The results showed that compound **B** had significant coagulant activity.

### 2.3. The Pro-Coagulant Effect of S. heptaphylla Extracts and Compound **B** In Vivo

#### 2.3.1. Effects on Plasma Coagulation Parameters

**Figure 4:**
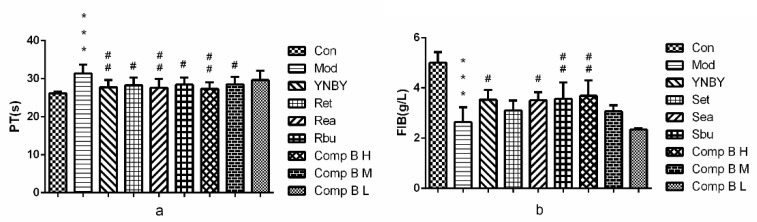
Effect on plasma coagulation parameters (**a**. PT; **b**. FIB, *n* = 8, ^***^ *p* < 0.001 vs. control group 0.001 < ^##^ *p* < 0.01, 0.01 < ^#^ *p* < 0.05 vs. model group).

In the model group, PT and FIB were significantly prolonged and decreased (*p* < 0.001 vs. control group), indicating that the heparin rats model was established successfully. In Figure 4. PT were shortened and the content of FIB was increased significantly in rats of the Set, Sea, Sbu groups compared with the model group (0.01 < *p* < 0.05 or 0.001 < *p* < 0.01). The effect of Sea on PT was even better than that of YNBY (0.01 < *p* < 0.001). Compound **B** (**M**, **H**) groups could short PT and increase the content of FIB effectively, the effect of compound **B H** was better than that of YNBY (0.01 < *p* < 0.001). Among the six groups (Set, Sea, Sbu, compound **B** (**H**, **M**, **L**), compound **B H** (0.16 g/kg) had the best pro-coagulant effect.

Blood coagulation results from a series of proteolytic reactions involving the step-wise activation of coagulation factors I—XII. Subsets of these factors can be activated by two distinct pathways, the extrinsic and the intrinsic pathway [24,25]. The APTT is commonly used for determining the overall efficiency of the intrinsic coagulation pathway, while PT is the screening test for the extrinsic coagulation pathway [26,27]. FIB is synthesized by the liver and can be hydrolyzed by the clotting enzyme to form peptide A and peptide B, eventually form the insoluble fibrin to stop bleeding [28]. Our experiments showed that Sea, Sbu and compound **B** could significantly shorten TT, PT, APTT, and increase the content of FIB, which indicated that compound **B** may be the main pro-coagulant active ingredient and promotes coagulation through the extrinsic and the intrinsic pathway, and promoting fibrin formation.

#### 2.3.2. Effects on Whole Blood Viscosity (WBV) and Plasma Viscosity (PV)

Hemorheology, used for diagnosis cardiovascular diseases in clinic, is related to blood flow and pressure, flow volume, and resistance of blood vessels, including WBV, PV, ESR and PCV [29,30,31]. In this paper, the hemorheology index were measured to study the mechanism of pro-coagulant effect of compound **B** in vivo. WBV and PV are closely related to ESR and PCV, which has been shown that PCV is the main factor influencing the plasma viscosity and there is a positive correlation between PCV and PV.

**Figure 5:**
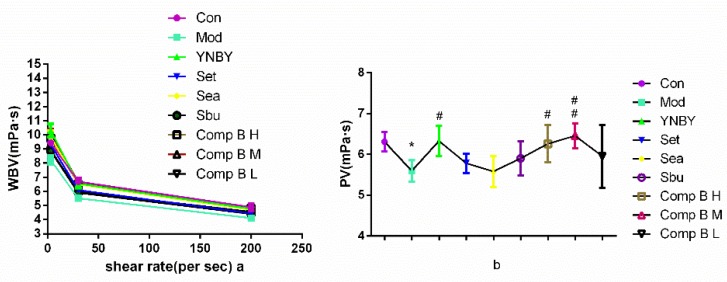
Effects of YNBY, Set, Sea, Sbu and compound B on WBV (**a**) and PV (**b**) (*n* = 8, 0.01 < ^*^ *p* < 0.05 vs. control group, 0.001 < ^##^ *p* <0.01, 0.01 < ^#^ *p* < 0.05 vs. model group).

In Figure 5, WBV and PV in the model group were significantly increased at all shear rates in the blood stasis (*p* < 0.001 vs. control group), indicating that the heparin rats model was established successfully. Set, Sea, Sbu and compound **B** (H, M) groups could significantly increase WBV and PV at all shear rates (*p* < 0.001, 0.001 < *p* < 0.01, *p* < 0.05 vs. model group). WBV was significantly increased in the compound **B** M group, the effect is similar to the YNBY group at all shear rates (*p* > 0.05). The effect of compound **B** (H, M) on increasing PV was similar to YNBY at low shear rate (*p* > 0.05).

#### 2.3.3. Effects of *S. heptaphylla* Extracts and Compound **B** on ESR and PCV

**Figure 6:**
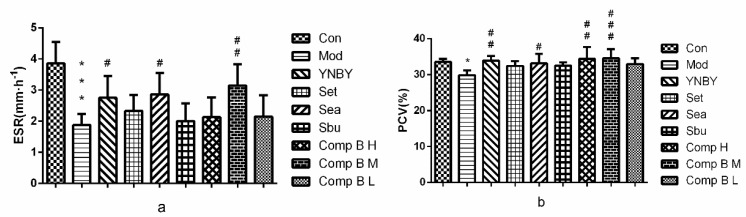
Effects of YNBY, Set, Sea, Sbu and compound **B** on ESR (**a**) and PCV (**b**) (*n* = 8, ^***^ *p* < 0.001, 0.01 < ^*^ *p* < 0.05 vs. control group, ^###^ *p* < 0.001, 0.001 < ^##^ *p* < 0.01, ^#^ *p* < 0.05 vs. model group).

In Figure 6, ESR and PCV in the model group were significantly lower than that of the control group (*p* < 0.001, *p* < 0.05), which suggested that the heparin rats model was established successfully. Set, Sbu and compound **B L** groups had no significant difference compared with model group. In Sea, compound **B M** and compound **B H** groups, ESR (*p* < 0.05, *p* < 0.01, *p* > 0.05, respectively) and PCV (*p* < 0.05, *p* < 0.001, *p* < 0.01, respectively) were significantly higher than that of the model group. The effects of compound **B M** were similar to YNBY.

The results showed that ESR and PCV of compound **B** (**M**, **H**) groups were significantly higher than that of the model group, meanwhile, compound **B** (**H**, **M**) groups could significantly increase WBV and PV compared with control group, which revealed that compound **B** had an pro-coagulant effect by regulating PCV and ESR.

#### 2.3.4. Effects of *S. heptaphylla* on Tail Bleeding Time

**Figure 7:**
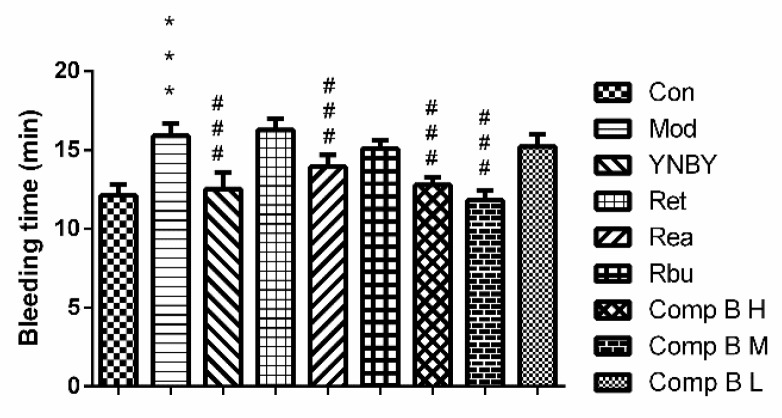
The effects of *S. heptaphylla* treatment on Bleeding Time (*n* = 8, ^***^ *p* < 0.001, ^###^ *p* < 0.001 vs. model group).

In order to explore the mechanism of compound **B** in promoting blood coagulation and hemostasis, the bleeding rat model was established by deep subcutaneous injection of heparin sodium salt (500 U/kg) to rats. The tail bleeding time of rats was the most vital parameter to evaluate the hemostatic activity of drugs. In Figure 7, the group of heparin (500 U/kg) significantly prolonged the bleeding time compared with control group (*p* < 0.001). YNBY (300 mg/kg) as a positive control could shorten bleeding time significantly (*p* < 0.001). Sea, compound **B M** and compound **B H** showed obvious influence on rat’s bleeding time (*p* < 0.001). The hemostatic effect of compound **B M** group was the best, which is equivalent to that of YNBY. In our research, sea and compound **B** (**M**, **H**) could significantly shorten the bleeding time, which maybe get benefit from increasing the content of FIB.

#### 2.3.5. TXB2 and 6-keto-PGF_1α_ in Serum

**Figure 8:**
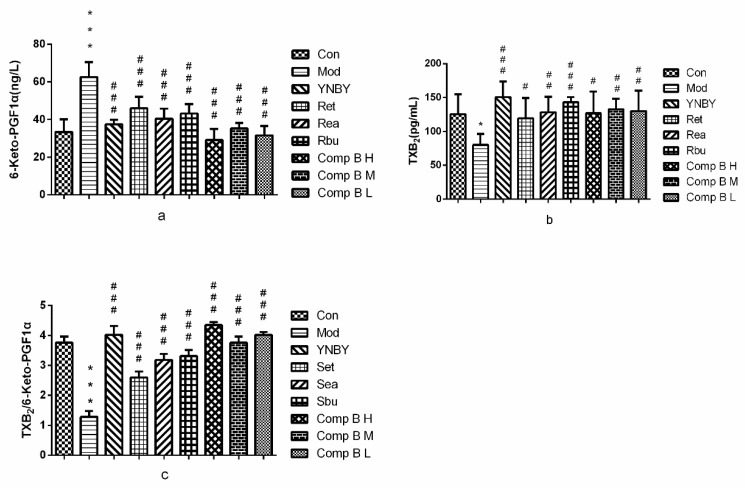
6-keto-PGF_1α_ (**a**) and TXB_2_ (**b**) levels, and TXB_2_/6-keto-PGF_1α_ (**c**) ratios in rats treated with YNBY, *S. heptaphylla* extracts and Compound **B** (*n* = 8, ^***^ *p* < 0.001, ^*^ *p* < 0.05 vs. control group, ^###^ *p* < 0.001, 0.001 < ^##^ *p* < 0.01, ^#^ *p* < 0.05).

Thromboxane A_2_ (TXA_2_) is potent vasoconstriction and platelet aggregation [32,33], which could stimulate platelet deformation and aggregation and cause the contraction of vascular smooth muscle. Prostacyclin (PGI_2_) could effectively inhibit platelet aggregation and blood vessel expansion. TXB_2_ and 6-Keto-PGF_1α_, which are mutually antagonistic, are the stable metabolites of TXA_2_ and PGI_2_, respectively [34]. The dynamic balance of TXB_2_/6-Keto-PGF_1α_ is important for the regulation of vessel wall intensity and regional flow. In Figure 8, the level of TXB2 in heparin model rats (80.22 ± 16.44 ng/L) was significantly lower than that of the control group (125.61 ± 29.25 ng/L) (*p* < 0.05), whereas the level of 6-keto-PGF_1α_ in heparin model rats (62.57 ± 7.99 ng/L) was significantly higher than that of control group (33.43 ± 6.70 ng/L) (*p* < 0.001), which indicated that the heparin rats model was established successfully. Set, Sea, Sbu and compound **B** (**H**, **M**, **L**) could significantly increase the level of TXB_2_ and decrease the level of 6-keto-PGF_1α_ in heparin model rats (*p* < 0.001, *p* < 0.01, *p* < 0.05). Meanwhile, Set, Sea, Sbu and compound **B** (**H**, **M**, **L**) could increase the ratio of TXB2/6-keto-PGF1αcompared with the model group (*p* < 0.001) and compound **B H** was better than that of YNBY group.

The results suggested that Set, Sea, Sbu and compound **B** (**H**, **M**, **L**) could significantly increase the level of TXB_2_ and decrease the level of 6-keto-PGF_1α_ in heparin model rats. In addition, all the experimental groups could increase the ratio of TXB_2_/6-keto-PGF_1α_ compared with the model group and compound **B H** group was even better than that of YNBY group. We speculated that the hemostasis and pro-coagulation effects of them maybe were associated with the regulation of TXA_2_ and PGI_2_.

#### 2.3.6. ET-1 and eNOS In Serum

**Figure 9:**
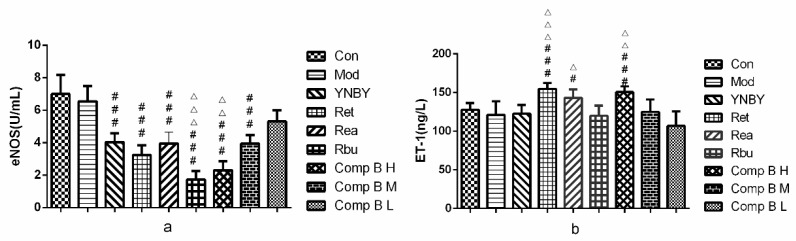
eNOS (**a**) and ET-1 (**b**) levels in rats treated with YNBY, *S. heptaphylla* extracts and Compound **B** (*n* = 8, ^###^ *p* < 0.001, 0.01 < ^#^ *p* < 0.05 vs. model group, ^△△△^ *p* < 0.001, 0.001 < ^△△^ *p* < 0.01, ^△^ *p* < 0.05 vs. YNBY).

In Figure 9, the level of ET-1 in heparin model rats (121.15 ± 17.38 μg/L) was lower than that of the control group (127.75 ± 8.65) (*p* > 0.05), whereas the level of eNOS in model rats (6.55 ± 0.92 U/L) was close to the control group (6.99 ± 1.18 U/L) (*p* > 0.05). The results showed that heparin had weaker effect on this the level of ET-1 and eNOS, so there was no significant difference between the model group and the control group. Set, Sea and compound **B H** could significantly up-regulate the level of ET-1 compared with model rats (*p* < 0.001, *p* < 0.05), and its effect was better than that of YNBY (*p* < 0.001, *p* < 0.01, *p* < 0.05). Set, Sea, Sbu and compound **B** (**M**, **H**) could significantly down-regulate the level of eNOS compared with the model group (*p* < 0.001), and the effect of Sbu and compound **B H** was better than that of YNBY (*p* < 0.001, *p* < 0.01). Among these groups, the effect of compound **B M** on up-regulating the level of ET-1 and down-regulate the level of eNOS was the best.

Our experimental results showed that Set, Sea and compound **B H** could significantly up-regulate the level of ET-1, which was better than that of YNBY. Set, Sea, Sbu and compound **B** (**M**, **H**) could significantly down-regulate the level of eNOS, and the effect of Sbu. Compound **B H** was better than that of YNBY. Thus, we speculated that the leaves of *S. heptaphylla* promoted pro-coagulant action by regulating the leaves of eNOS and ET-1.

## 3. Materials and Methods

### 3.1. General

^1^D-NMR spectra were recorded on a Bruker-AM-400 spectrometer (Billerica, MA, USA) tetramethylsilane (TMS) used as an internal standard. Electron ionization-mass spectrometry (EI-MS) spectra were obtained by a Thermo Trace DSQ II-mass spectrometer (Thermo Fisher Scientific Inc., MA, USA). HF6000 Semi-Automated Coagulation Analyzer (Chinese Prescription Medical Instrument Co., Ltd., Jinan, Shandong, China). TGL-16 high speed centrifuge (Zhongda Instrument Factory, Jintan, Jiangsu, China). Sephadex LH-20 (Pharmacia, Sweden); silica gel (100–200, 200–300 mesh: Qingdao Marin Chemical Co., Ltd., Qingdao, China).

#### 3.1.1. Drugs and Reagents

Injection breviscapine was obtained from Hang Sheng Pharmaceutical Co., Ltd., (Hunan, China). PT, APTT, TT and FIB assay kits were purchased from Shanghai Sun Biotech Co., Ltd. (Shanghai, China). TXB_2_ ELISA Kit, 6-keto-PGF_1α_ ELISA Kit, eNOS ELISA Kit and ET-1 ELISA Kit were purchased from NanJing Jiancheng Bioengineering Institute (NanJing, China). Yunnanbaiyao (YNBY) was a product of Yunnanbaiyao Group Co., Ltd. (Kunming, China). Heparin Sodium Injection was obtained from Maanshan pharmaceutical Co. Ltd. (Anhui, China).

#### 3.1.2. Plant Material

*Scheffleraheptaphylla* fresh leaves were collected from Quanzhou (118°36’ E, 24°58’ N), Fujian Province, China, in April 2017, and the plant was identified by Prof. Changqin Li in Henan University (Kaifeng, Henan, China). A voucher specimen (No.20170502) was deposited in Henan University.

#### 3.1.3. Animal

Male and female Sprague-Dawley (SD) rates (200–220 g) and rabbits (2–2.5 kg) were provided by the Experimental Animal Center of Henan Province (Zhengzhou, Henan, China), They were housed in cages unrestricted access to food and water with a constant temperature (ca.25 ± 1 °C) and humidity(ca.60 ± 2%) and with a 12 h light/dark cycle. The animal procedures were approved by the Ethical Committee in accordance with ‘Institute ethical committee guidelines’ for Animal Experimentation and Care (The study obtained ethical clearance from the Ethics Committee of College of Medical, Henan University (NO: 2018-36)). All animals were underwent 7 days’ adaption, and before experiments, food was not given for 12 h and water was available at any time.

### 3.2. Experimental

#### 3.2.1. Extraction and Isolation

Dried leaves of (10 kg) were extracted by petroleum ether (50 L) for degreasing, 5 times, every time for 3 days, at room temperature. After the petroleum ether was volatilized, the residues were extracted three times with 75% (*v*/*v*) ethanol (50 L) at room temperature, every time for 3 days. The solution was evaporated under reduced pressure to obtain an ethanol extract (1.3 kg). The residue was suspended in distilled water and then extracted with petroleum ether, ethyl acetate (EtOAc) and *n*-butanol (*n*-BuOH), respectively. The solutions were evaporated under reduced pressure to yield an EtOAc extract (800 g) and *n*-BuOH extract (1 kg).

The EtOAc extract was subjected to silica gel (200–300 mesh) column chromatography and eluted with CH_2_Cl_2_-MeOH gradient (100:0, 100:1, 50:1, 20:1, 10:1, 5:1 and 1:1) to give 6 fractions (F1–F5). F3 was separated on a silica gel H with CH_2_Cl_2_-MeOH gradient solvent with an ODS open gel column by gradient mixtures of MeOH-H2O (40:60, 20:80, 100:0) to yield system (100:1, 50:1, 20:1, 10:1) to yield compound **A** (12 g) and then F3.3 purified by eluted compound **B** (15g). F4 was separated by chromatography over silica gel by elution with a CH_2_Cl_2_–MeOH gradient (100:0, 100:1, 50:1, 20:1, 10:1, 5:1 and 1:1) to afford four subfractions (F4.1, F4.2, F4.3 and F4.4). F4.2 was further subjected to fractionated on SephadexLH-20 (MeOH), and further again subjected to SephadexLH-20 (MeOH) to yield compound **C** (22 g).

#### 3.2.2. Plasma Anticoagulation Assay In Vitro

Blood samples were drawn from the auricular veins of rabbits. After collection, the blood was decalcified by sodium citrate to prevent blood clotting, the serum was separated from the plasma by centrifugation at 3000 rpm at 5 °C for 15 min [41] to assay activated partial thromboplastin time (APTT), prothrombin time (PT), thxrombin time (TT) and fibrinogen (FIB) in vitro.

PT and APTT assays

APTT and PT were determined. In brief, 25 μL of samples were mixed with serum (50 μL) and APTT assay reagent (50 μL), incubated at 37 °C for 5 min, and then 25 mM CaCl_2_ (100 μL) was added. The clotting times were recorded as APTT. For the PT assays, 25 μL of samples were mixed with serum (50 μL), incubated at 37 °C for 3 min. PT assay reagent (50 μL), incubated at 37 °C for 10 min, was added and clotting time was recorded.

TT and FIB assays

Samples of (50 μL) was mixed with 200 μL of plasma, incubated for 3 min at 37 °C, then 200 μL of reagent of TT was added and the clotting time was recorded as TT. Samples of (100 μL) was mixed with plasma (200 μL), then buffer (700 μL) was added to obtain mixed solution incubated at 37 °C for 3 min, then 100 μL of all the enzyme solution was added and the content of FIB was recorded.

In the above tests, blank solvent was used as the blank control group and breviscapine, YNBY was used as the positive control group. PT, APTT, TT and FIB tests were measured by a Semi-Automated Coagulation Analyzer.

#### 3.2.3. Biological Activity Assays In Vivo

Experimental model and drug administration

Rats were randomly divided into nine groups with eight animals in each group, whose gender was equally distributed throughout groups. The control group: rats were given blank solvent at the same volume. The model group: rats were given blank solvent at the same volume. The YNBY group: rats were given 300 mg/kg YNBY. Groups 4–6 were model + extracts: rats were given Set (1 g/kg), Sea (1 g/kg) and Sbu (1 g/kg), respectively. Groups 7–9 were model + compound **B** (**H**, **M**, **L**): rats were given compound **B** (0.16 g/kg, 0.08 g/kg and 0.04 g/kg, respectively).

The control group rats were injected with 0.9% (*w*/*v*) NaCl saline solution, and all other group rats were injected subcutaneously with heparin (500 U/kg) 5 min after the last administration. Rats were anesthetized with 10% chloral hydrate (0.4 mL/100g) 50 min after the injection of heparin, and blood samples were collected after testing bleeding time.

#### 3.2.4. Bleeding Time Assay

Bleeding time was measured after the injection of chloral hydrate immediately. The tail bleeding model was conducted based on the previous methods with slight modifications [42]. The tails of rats were transected with a sterile razor blade the site that 5 mm apart from the tip. The wound was gently wiped with filter paper every 30 s. The bleeding time was from the start of transection to bleeding cessation. The wound stopped bleeding after 30 s without bleeding. Stop counting bleeding if the bleeding time was more than 30 min.

### 3.3. Collection of Blood Samples

Rats were anesthetized with 10% chloral hydrate (0.4 mL/100 g) 50 min after the last injection of heparin, and blood was drawn from the abdominal aortas. Blood was collected by disposable vein infusion needles. 1 mL of blood sample was collected into centrifuge tube and centrifuged at 3000 r/min for 15 min to get serum, which was used for detection of eNOS, ET-1, 6-keto-PGF_1α_, and TXB_2_. 2 mL of the blood sample was taken into plain tubes containing heparin for the detection of the blood viscosity parameters, including blood viscosity of low shear (BVL), blood viscosity of medium shear (BVM), blood viscosity of high shear (BVH) and PV. 3 mL of blood sample was collected into vacuum vasculature with the anticoagulant heparin sodium to measure the indexes of PT and TT. 2 mL of blood sample was kept into vacuum blood tube with the anticoagulant citrate sodium to detect the indexes of PCV and ESR.

## 4. Statistical Analysis

All the experimental data were expressed as mean ± standard deviation (SD). Statistical analysis was performed with the SPSS19.0. Comparison between any two groups was evaluated using one-way analysis of variance (ANOVA).

## 5. Conclusions

*S. heptaphylla* extracts and compound **B** possessed remarkable pro-coagulation properties in heparin model rats induced by subcutaneous injection of heparin. Compound **B** may be the main active ingredient of *S. heptaphylla* responsible for pro-coagulation, and we found that compound **B** (**H**, **M**) in doses of 160 mg/kg and 80 mg/kg could had effectively pro-coagulant effect in a heparin model rats. This property may be associated with its activation of blood flow, pro-coagulation activity, the regulation of active substances in vascular endothelium and regulation of the balance between TXA_2_ and PGI_2_.

## Abbreviations

APTT	activated partial thromboplastin time
PT	prothrombin time
TT	thrombin time
FIB	plasma fibrinogen
6-keto-PGF_1α_	6-keto prostaglandin F_1α_
eNOS	nitric oxide synthase
TXB_2_	thromboxane B_2_
ET-1	endothelin-1
ESR	blood sedimentation
PCV	hematocrit
WBV	whole blood viscosity
PV	plasma viscosity
